# Supplementary material for: Content-rich biological network constructed by mining PubMed abstracts
Source: BMC Bioinformatics. 2004 Oct 8;5:147. doi: 10.1186/1471-2105-5-147 (PMC528731; doi:10.1186/1471-2105-5-147)
Supplement: Additional File 2 — The original results of the above study (non-essential files are deleted to keep the file size under the limit set by BMC bioinformatics). [file 1471-2105-5-147-S2.bz2 › chilibotAdditionalFile2/dip20/638ID8114095E11025/html/TRXA_TRXB.html]

 


 **TRXA** and **TRXB** 
  
Found 679 abstracts in PubMed, retrieved 20.  
 

 What does Google say? 
 PDF only 
| .edu only 

---

**Interactive relationship** (e.g. stimulation, inhibition, etc)

**Inhibitory relationship**- Peroxiredoxin 3 Prdx3 is a mitochondrial member of the antioxidant family of thioredoxin  [ **TRXA** ]  peroxidases that uses mitochondrial thioredoxin  [ **TRXA** ]  2 Trx2  [ **TRXB** ]  as a source of reducing equivalents to scavenge hydrogen peroxide H 2 O 2.  Ref: 12861054 Mol Cancer Res, 2003
**Neutral relationship**

**Non-interactive relationship** (e.g. studied together, co-existance, homology, etc.)

- Necrotic tumors were more often immunopositive for Trx  [ **TRXA** ]  p = 0.001 and TrxR  [ **TRXB** ]  p = 0.02 and AOE expression was generally higher in mitotically active tumors.  Ref: 12744469 Brain Pathol, 2003
- Western blot showed that temocapril enhanced cytosolic redox regulatory protein TRX  [ **TRXA** ]  expression, but neither mitochondrial TRX2  [ **TRXB** ]  nor antioxidant enzymes, such as copper zinc superoxide dismutase Cu Zn SOD or manganese superoxide dismutase Mn SOD expression, was increased by the preconditioning treatment.  Ref: 12870672 Mol Cell Biochem, 2003
- Within diffuse astrocytomas only Trx  [ **TRXA** ]  p = 0.0001 and TrxR  [ **TRXB** ]  p= 0.04 significantly associated with increased malignancy grade.  Ref: 12744469 Brain Pathol, 2003
- We studied the expression of antioxidant enzymes AOEs and related proteins manganese superoxide dismutase MnSOD, thioredoxin  [ **TRXA** ]  Trx  [ **TRXA** ] , thioredoxin  [ **TRXA** ]  reductase TrxR  [ **TRXB** ] , and the catalytic GLCL c and regulatory GLCL r subunits of glutamate cysteine ligase gamma glutamylcysteinesynthetase in 433 astrocytomas.  Ref: 12744469 Brain Pathol, 2003
- Diffuse astrocytomas showed more intense staining for Trx  [ **TRXA** ]  p = 0.002, TrxR  [ **TRXB** ]  p = 0.004, GLCL c p = 0.001, GLCL r p = 0.04 and MnSOD p = 0.01 than pilocytic astrocytomas.  Ref: 12744469 Brain Pathol, 2003
- Expression of MnSOD was found in 91%, Trx  [ **TRXA** ]  in 46%, TrxR  [ **TRXB** ]  in 66%, GLCL c 73% and GLCL r in 89% of the cases.  Ref: 12744469 Brain Pathol, 2003
- RESULTS Temocapril enhanced cytosolic redox regulatory protein TRX  [ **TRXA** ]  expression, but neither mitochondrial TRX2  [ **TRXB** ]  nor antioxidant enzymes, such as copper zinc superoxide dismutase Cu Zn SOD or manganese superoxide dismutase Mn SOD expression, was up regulated by the preconditioning treatment.  Ref: 12882710 Zhonghua Nei Ke Za Zhi, 2003
- Data from the present data further showed that while Trx  [ **TRXA** ]  and Trx  [ **TRXA** ]  reductase TrxR  [ **TRXB** ]  in cytoplasm decrease with age in AL fed rats, CR prevents these decreases.  Ref: 12742531 Exp Gerontol, 2003
